# Comparative analysis of integument transcriptomes identifies genes that participate in marking pattern formation in three allelic mutants of silkworm, *Bombyx mori*

**DOI:** 10.1007/s10142-019-00708-w

**Published:** 2019-09-02

**Authors:** Xin Ding, Junxia Liu, Xiaoling Tong, Songyuan Wu, Chunlin Li, Jiangbo Song, Hai Hu, Duan Tan, Fangyin Dai

**Affiliations:** State Key Laboratory of Silkworm Genome Biology, Key Laboratory of Sericultural Biology and Genetic Breeding, Ministry of Agriculture and Rural Affairs, College of Biotechnology, Southwest University, Chongqing, 400715 China

**Keywords:** *Bombyx mori*, Allelic mutants, Integument, Transcriptome, Pigment, Marking pattern

## Abstract

The diversity markings and pigment patterns in insects are outcomes of adaptive evolution. The elucidation of the molecular mechanism underlying variations in pigment patterns may improve our understanding of the origin and evolution of these spectacular diverse phenotypes. Melanin, ommochrome, and pteridine are the three main types of insect pigments, and the genes that directly participate in pigment biosynthesis have been extensively studied. However, available information on gene interactions and the whole pigment regulatory network is limited. In this study, we performed integument transcriptome sequencing to analyze three larval marking allelic mutants, namely, multi lunar (*L*), *L*^*C*^, and *L*^*Ca*^, which have similar twin-spot markings on the dorsal side of multiple segments. Further analysis identified 336 differentially expressed genes (DEGs) between *L* and Dazao (wild type which exhibits normal markings), 68 DEGs between *L*^*C*^/+ and +^*LC*^/+^*LC*^, and 188 DEGs between *L*^*Ca*^/+ and +^*LCa*^/+^*LCa*^. Gene Ontology (GO) analysis indicated a significant DEG enrichment of the functional terms catalytic activity, binding, metabolic process, and cellular process. Furthermore, three mutants share six common enriched KEGG pathways. We finally identified eight common DEGs among three pairwise comparisons, including *Krueppel*-like factor, TATA-binding protein, protein patched, UDP-glycosyltransferase, an unknown secreted protein, and three cuticular proteins. Microarray-based gene expression analysis revealed that the eight genes are upregulated during molting, which coincides with marking formation, and are significantly differentially expressed between marking and non-marking regions. The results suggest that the eight common genes are involved in the construction of the multiple twin-spot marking patterns in the three mutants.

**Electronic supplementary material:**

The online version of this article (10.1007/s10142-019-00708-w) contains supplementary material, which is available to authorized users.

## Introduction

Insects are the most diverse and abundant group, and more than 1 million species are distributed around the world. Their success relies on the variety of traits developed during their evolution, including the formation of various markings and pigment patterns. The easily observable intra- and interspecific highly diversified body color patterns of insects are good system to studying the genetic mechanism of phenotypic evolution. In addition, diversified body color patterns are benefit for insects, such as avoid predators, mate choice, immunity, thermoregulation, and UV protection (Kronforst et al. 2012; Takahashi 2013; Wittkopp and Beldade 2009; Wittkopp et al. 2003). Therefore, it is essential to elucidate the molecular genetic mechanism of the occurrence and differentiation of insect coloring.

Melanin, ommochrome, and pteridine are the three major types of insect pigments. The genes involved in the pigments syntheses had been well identified in insects and are conserved in different species. However, in addition to the genes that directly participate in pigment synthesis, recent studies have shown that many regulatory genes are also involved in the regulation of the expression of pigment synthesis genes that determine coloring patterns. The *Wnt* family gene is revealed related to the establishment of color patterns of fruit fly, butterfly, and silkworm (Carroll et al. 1994; Gallant et al. 2014; Koshikawa et al. 2015; Martin et al. 2012; Martin and Reed 2014; Mazo-Vargas et al. 2017; Yamaguchi et al. 2013). The diversification of *cis*-regulatory elements of *wg* (*wingless*) allows regulation of its expression, resulting in the spotting pattern in *Drosophila guttifera* and *Bombyx mori* (Koshikawa et al. 2015; Yamaguchi et al. 2013). *WntA*, another member of the *Wnt* family, is also related to the establishment of black wing patterns in *Heliconius* genus through the diversification of *cis*-elements (Gallant et al. 2014; Martin et al. 2012; Martin and Reed 2014; Mazo-Vargas et al. 2017). Except the *Wnt* genes, there are many other regulatory genes in different species. In *Drosophila*, *optomotor-blind* (*omb*), *bric a brac* (*bab*), *abdominal-B* (*Abd-B*), *doublesex* (*dsx*), *Distal-less* (*Dll*), and *Engrailed* (*en*) have been shown to regulate expression of pigment synthesis genes either directly or indirectly (Massey and Wittkopp 2016; Tong et al. 2014). In butterfly, *Optix*, *Ubx*, *Antp*, *Dll*, *E75*, *spalt*, and *cortex* genes have been shown that associated with the formation of eye spots on wings (Futahashi et al. 2012; Tong et al. 2014). In silkworm, upregulation of *apontic-like* gene and Toll signaling pathway receptor *Spätzle3* promotes the pigmentation of silkworm larvae (KonDo et al. 2017; Yoda et al. 2014).

**Fig. 1:**
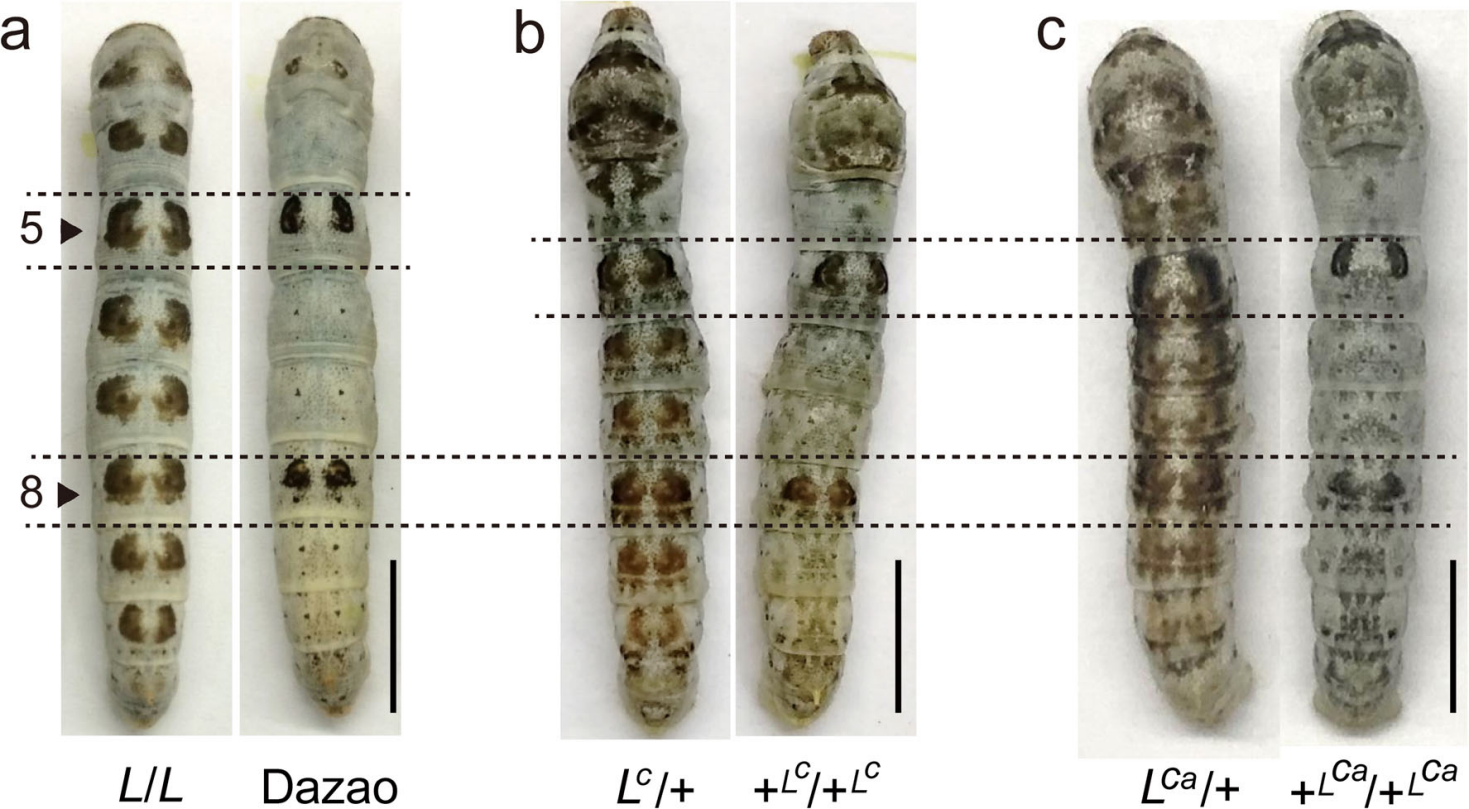
The phenotype of *L*-type mutant and wild type at HCS of four-instar larval stage. **a** Homozygous *L* mutant and wild-type strain Dazao. *L* is an inbred line of Dazao that was generated after several years of crossing. **b** Heterozygous *L*^*C*^ mutant and +^*Lc*^/ +^*Lc*^. The *L*^*C*^ mutant possesses triangulated markings on the dorsal of multiple segments. **c** Heterozygous *L*^*Ca*^ mutant and the +^*LCa*^/+^*LCa*^. The *L*^*Ca*^ mutant possesses squared markings with irregular edge

Identification of these genes and regulatory elements enriches our understanding of insect pigment regulatory mechanisms; it also reveals the complexity of the associated regulatory network. In addition, compared with the extensive studies that focus on the adult wing spot pigmentation of fruit flies and butterflies, investigations on the diversity of noticeable larval marking pigmentation remains limited (Shirataki et al. 2010). Over 100 years of genetic studies on the lepidopteran *Bombyx mori* have accumulated nearly 200 strains of mutants exhibiting variations in body color and stripes, thereby making it an ideal model for research on the molecular mechanism of complex color patterns in caterpillars. In silkworm, multi lunar (*L*), *L*^*C*^, and *L*^*Ca*^ are three spontaneous mutations which have similar twin-spot markings on the dorsal multiple segments (Fig. 1); previous classical genetic analysis revealed that the three mutants were allelic mutations. Similar phenotypes and allele mutations suggest that these three mutants may have common regulatory basis. Fujiwara et al. revealed that *Wnt1* gene was responsible for *L* mutant by localized cloning (Yamaguchi et al. 2013)*.* In this study, we performed RNA-seq of the three similar phenotypic mutants to identify more candidate genes that are involved in the formation of marking patterns of the *L*-type mutants, as well as provide a reference for understanding the genetic basis of markings formation in caterpillar.

## Results

### Library construction and overview of RNA-seq

The color pattern of silkworm larva is reformed at every molting period, prompting us to select the onset of molting (head capsule slippage, HCS) stage of the 4th molting for RNA-seq to analyze the expression levels of genes that are related to pigmentation. Three similar phenotypic mutants (*L*, *L*^*C*^, and *L*^*Ca*^) were used in this study. The markings of the *L* mutant are crescent in shape, whereas that of *L*^*C*^ mutants are more triangulated and that of *L*^*Ca*^ are generally square with irregular edges. These similarities in phenotype suggest that the three mutants may have the same molecular basis in the formation of marking patterns. *L* is a near isogenic line of wild-type Dazao, so we selected and used *L* and Dazao for pairwise comparison. The homozygous embryo of *L*^*C*^ and *L*^*Ca*^ is lethal, and twin-spot markings are observed only on the 5th and 8th segments in both +^*LC*^/+^*LC*^ and +^*LCa*^/+^*LCa*^, which are generated by heterozygous *L*^*C*^ and *L*^*Ca*^ inbred lines, respectively (Fig. 1). Thus, we selected +^*LC*^/+^*LC*^ and +^*LCa*^/+^*LCa*^ as controls of heterozygous *L*^*C*^ and *L*^*Ca*^. Finally, a total of 18 transcriptome libraries containing three pairwise comparisons were constructed, including three mutants and the corresponding controls. Three biological duplicates of each sample were prepared and then sequenced on an Illumina HiSeq 2000 platform.

**Fig. 2:**
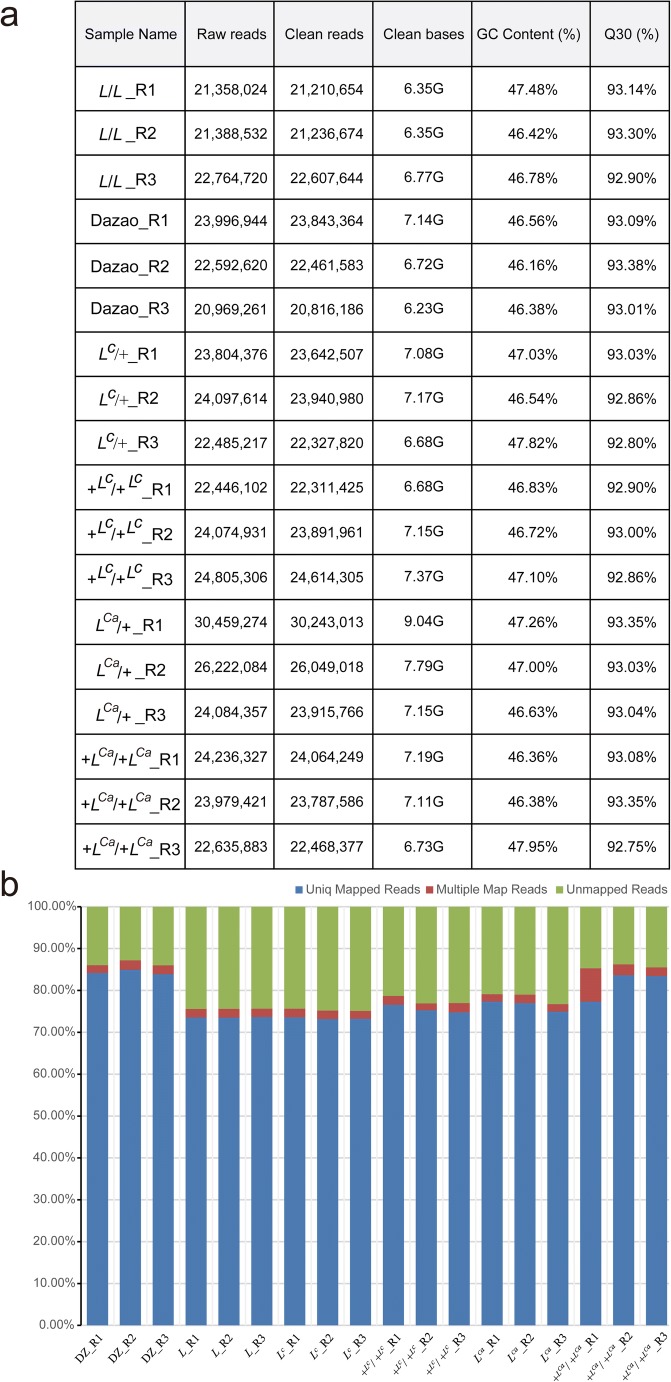
Overview of RNA-seq. **a** The numbers at the end of samples name represent biological replicates of every strain. Q30 represent the sequencing error rate *<* 0.1%. G represents gigabase. **b** Comparison efficiency statistics. The blue column represents unique mapped reads, the red column indicated multiple mapped reads, and the green column shows the unmapped reads

A total of 126.70-gigabase (Gb) clean reads were generated, and the data size of each sample is approximately 6.23 to 9.04 Gb, with a Q30 percentage (percentage of sequences with sequencing error rates < 1‰) varied between 92.75 and 93.38%. The clean reads of each sample were mapped to the reference genome of the silkworm, and the mapped efficiency of the samples varied from 75.10 to 87.21% (Fig. 2). The obtained mapped reads were assembled and compared with the original annotation information of the reference genome, which identified a total of 1225 novel transcripts (Online Resource 1). The nucleotide sequences of new transcripts were blast in databases including NR, GO, Swiss-Prot, COG, KOG, KEGG, and Pfam. A total of 1111 new genes were finally annotated, and the number of new genes annotated in each database is shown in Table 1.

**Table 1:** Number of new genes annotated in databases

Annotated databases	New gene number
COG	189
GO	530
KEGG	307
KOG	534
Pfam	606
Swiss-Prot	458
eggNOG	995
nr	1082
All	1111

### Analysis of differentially expressed genes

Based on the transcriptomes of three *L*-type multiple twin-spot marking pattern mutants and their control, we performed differentially expressed gene (DEG) analysis using edgeR (Robinson et al. 2010) in BMKcloud (http://www.biocloud.net) to identify the related genes involved in *L*-type marking pattern formation. Fragments per kilobase of transcript per million fragments mapped (FPKM) was used to calculate abundance of transcripts of each gene. Based on the 18 transcriptome libraries, a total of 12,095 expressed genes were identified and the FPKM values were calculated (Online Resource 2). Significant differential expression was considered to exist when the log_2_ fold change (logFC) of the mutant and control ratio is > 1 at a threshold of *P* value < 0.05. After analysis, 336 significantly DEGs were detected between *L* and Dazao, of which 189 were upregulated and 147 were downregulated; 68 significantly DEGs were detected between *L*^*C*^/+ and +^*LC*^/+^*LC*^, which consisted of 51 upregulated and 17 downregulated genes; and 188 DEGs were identified between *L*^*Ca*^/+ and +^*LCa*^/+^*LCa*^, comprising 120 upregulated and 68 downregulated genes (Table 2 and Online Resource 2).

**Table 2:** DEG analysis of the three pairwise comparisons

	Total DEGs	Upregulated	Downregulated
*L*/*L* VS Dazao	336	189	147
*L*^*C*^/+ VS +^*LC*^/+^*LC*^	68	51	17
*L*^*Ca*^/+ VS +^*LCa*^/+^*LCa*^	188	120	68

**Fig. 3:**
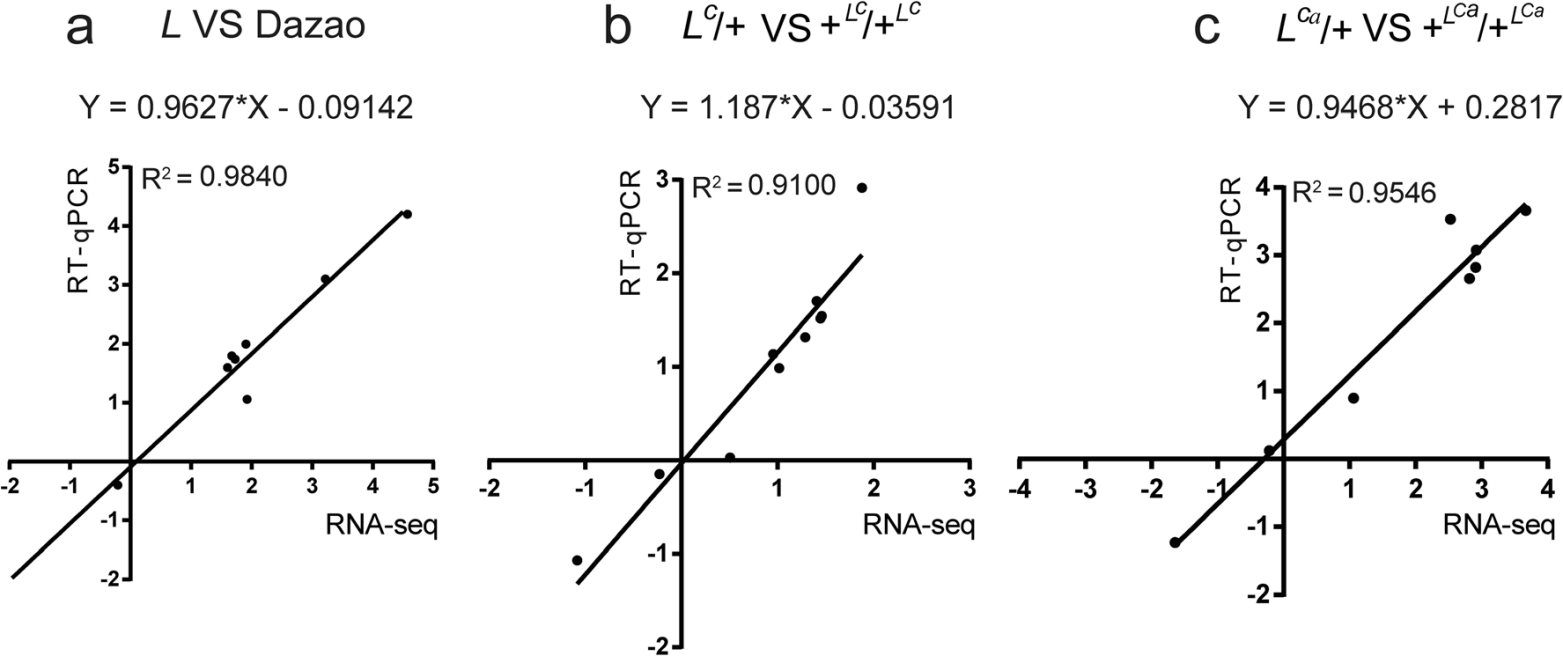
Comparison of gene expression values obtained by RNA-seq and real-time fluorescence quantitative PCR (RT-qPCR). The *x*-axis shows the log_2_FC from RNA-seq, the *y*-axis depicts the same values from RT-qPCR using the 2^−ΔΔc(t)^ algorithm. The *R*^2^ values show the correlation ratio between RNA-seq and RT-qPCR. **a** The logRT_2_FC of *L* mutant comparison of the wild-type Dazao. **b** The log_2_FC of heterozygous *L*^*C*^ mutant and the wild-type +^*LC*^/+^*LC*^. **c** The log_2_FC of heterozygous *L*^*Ca*^ mutant and the wild-type +^*LCa*^/+^*LCa*^

To validate our RNA-seq data, 12 DEGs were selected based on their up- and downregulated fold change values and assessed by RT-qPCR analysis. To intuitively compare RNA-seq data and RT-qPCR data, the gene expression levels generated by RT-qPCR were changed to log_2_ (fold change), which were then used in RNA-seq data analysis. The fold changes of the selected genes coincided with our RNA-seq data, indicating the reliability of our RNA-seq results (Fig. 3).

### Gene Ontology (GO) and KEGG pathway enrichment analysis of DEGs

**Fig. 4:**
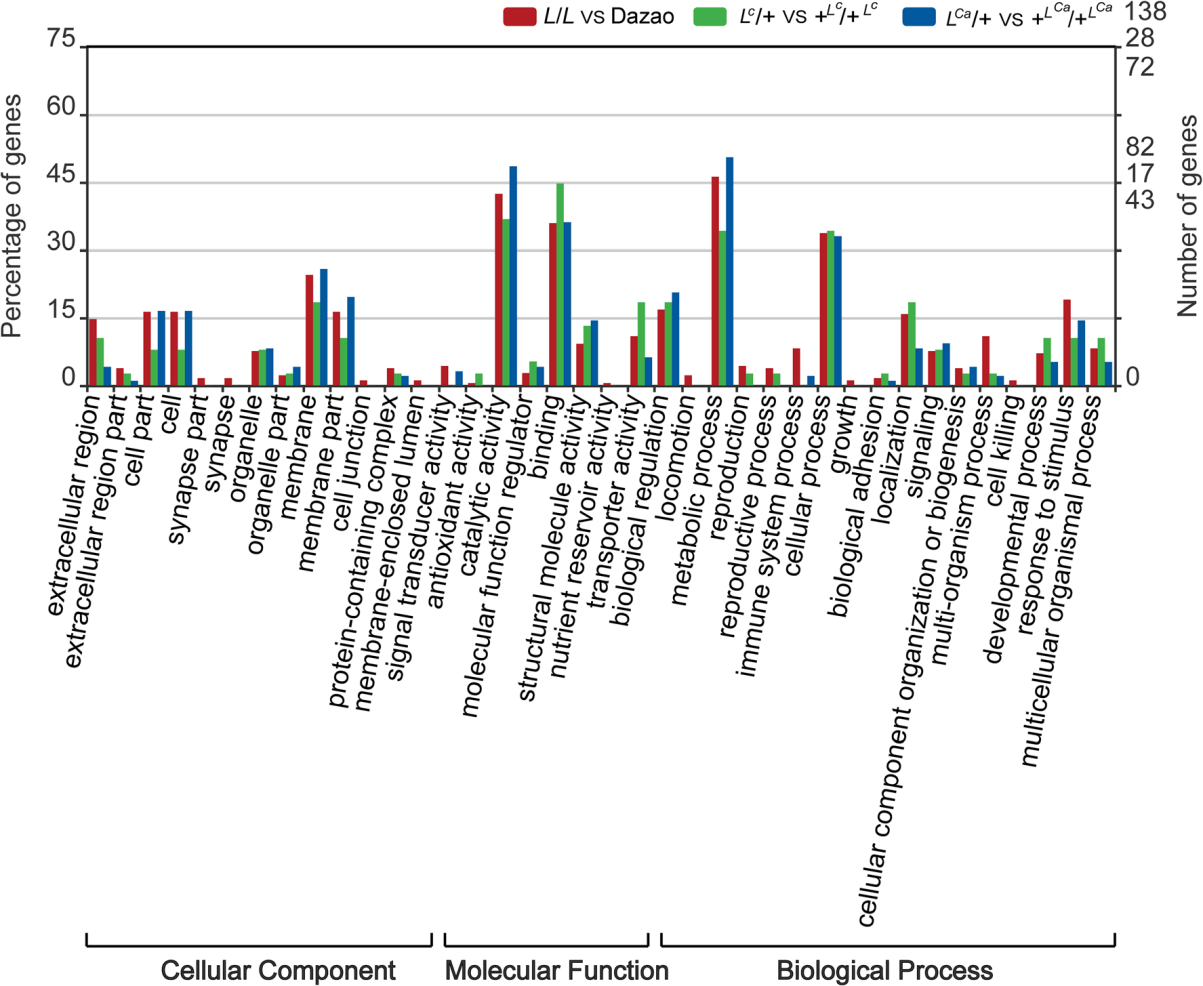
GO analysis of DEGs in the three pairwise comparisons. The annotated DEGs were classified into the functional categories of cellular component, molecular function, and biological process. Various colors represent different pairwise comparisons

**Fig. 5:**
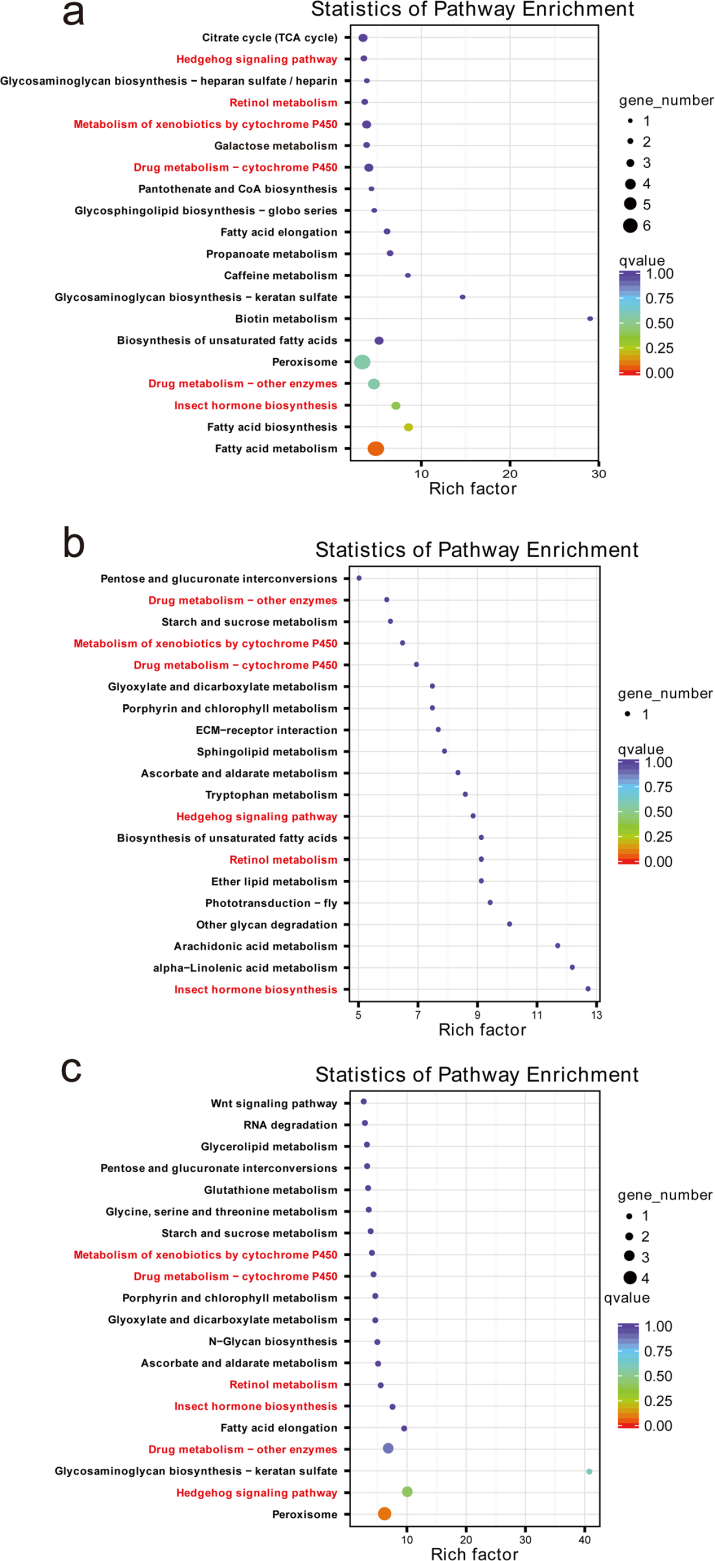
KEGG pathway enrichment analysis of DEGs in the three pairwise comparisons. **a***L*/*L* VS Dazao. **b***L*^*C*^/+ VS +^*LC*^/+^*LC*^. **c***L*^*Ca*^/+ VS +^*LCa*^/+^*LCa*^. The *x*-axis indicates the enrichment factor. The *y*-axis shows different pathways

To assess the molecular function of DEGs in the three mutant-control pairwise comparisons (*L*/*L* VS Dazao, *L*^*C*^/+ VS +^*LC*^/+^*LC*^, *L*^*Ca*^/+ VS +^*LCa*^/+^*LCa*^), GO categories and KEGG pathway enrichment analysis were performed. GO analysis divided the DEGs into three functional groups: biological process, cellular component, and molecular function (Fig. 4). The most significantly enriched terms in the three mutants included catalytic activity, binding, metabolic process, and cellular process. Then, the DEGs were mapped to reference pathways in the KEGG database, and the significantly enriched KEGG terms are shown in Fig. 5. These results show that the three mutants share six common enriched KEGG pathways, including hedgehog signaling pathway, retinol metabolism, metabolism of xenobiotics by cytochrome P450, drug metabolism-cytochrome P450, drug metabolism-other enzymes, and insect hormone biosynthesis (red in Fig. 5). The hedgehog signaling pathway is related to body plan and segment determination (Carballo et al. 2018; Nusslein-Volhard and Wieschaus 1980). In butterfly, the hedgehog signaling pathway has been shown to play a role in the establishment of eyespot patterns (Keys et al. 1999; Tong et al. 2012).

### The common DEGs are related to the multiple twin-spot marking formation in the three mutants

**Fig. 6:**
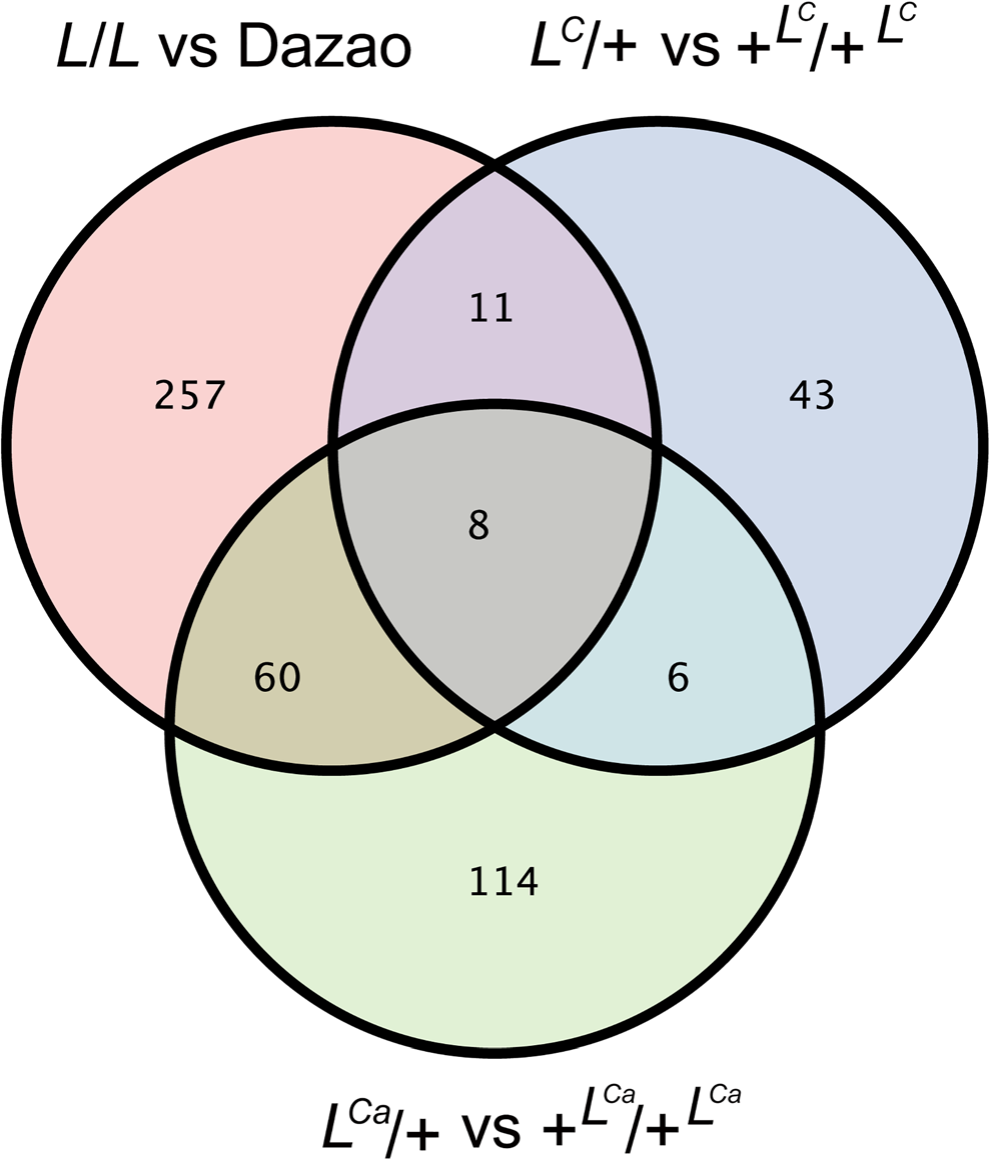
Venn diagram of DEGs. The eight overlapping genes are common to the three pairwise comparisons. The numbers 257, 43, and 114 represent the specific number of DEGs detected in the *L*, *L*^*C*^, and *L*^*Ca*^ mutants

To identify major DEGs linked to multiple twin-spot marking formation in the three similar phenotype mutants, a Venn diagram was constructed (Fig. 6). The results show eight DEGs that are common among the three mutants (Table 3). Among these eight DEGs, *BGIBMGA008464* (secreted protein), *BGIBMGA01382*8 (TATA-binding protein), *NewGene6331* (patched protein), *BGIBMGA014622* (UDP-glycosyltransferase), *BGIBMGA010500* (cuticular protein), *BGIBMGA006120* (*Krueppel*-like factor), and *NewGene_4929* (cuticular protein) are upregulated in the three mutants; *BGIBMGA013915*, which encodes a RR-2 motif cuticular protein, is downregulated in the three multiple twin-spot markings mutants.

**Table 3:** Common DEGs in the three mutants

Gene	Annotation	*L*/*L* VS Dazao	*L*^*C*^/+ VS +^*Lc*^/+^*Lc*^	*L*^*Ca*^/+ VS +^*Lca*^/+^*Lca*^
*BGIBMGA010500*	Cuticular protein	Up	Up	Up
*NewGene4929*	Cuticular protein	Up	Up	Up
*BGIBMGA006120*	*Krueppel*-like factor	Up	Up	Up
*BGIBMGA013828*	TATA-binding protein	Up	Up	Up
*NewGene6331*	Patched protein	Up	Up	Up
*BGIBMGA014622*	UDP-glycosyltransferase	Up	Up	Up
*BGIBMGA008464*	Unknown secreted protein	Up	Up	Up
*BGIBMGA013915*	RR2 cuticular protein	Down	Down	Down

**Fig. 7:**
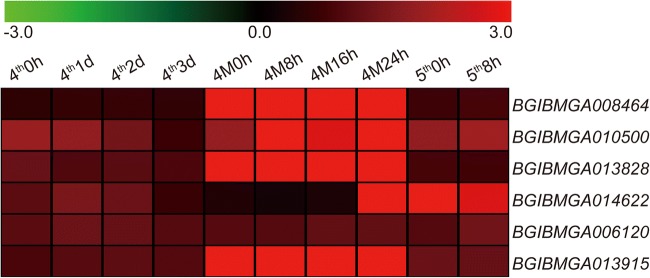
Expression microarray data analysis of the common DEGs in the 4th instar period of the silkworm. The color bar represents gene expression levels, red means high expression, and green represents low expression. The columns represent 10 stages from 4th instar to 5th instar. 4th0h, the start of 4th instar; 4th1d, the first day of 4th instar; 4th2d, the second day of 4th instar; 4th3d, the third day of 4th instar; 4M0h, start of 4th molting; 4M8h, the 8th hour of 4th molting; 4M16h, the 16th hour of 4th molting; 4M24h, the 24th hour of 4th molting; 5th0h, the start of 5th instar; 5th 8 h, the 8th hour of the 5th instar

To further analyze the relationship between the common genes and marking formation, we analyzed expression microarray data of the eight DEGs in the 4th instar period of the silkworm. Two of the 8 genes have no microarray data. The other 6 genes, *BGIBMGA008464*, *BGIBMGA010500*, *BGIBMGA013828*, *BGIBMGA014622*, *BGIBMGA006120*, and *BGIBMGA013915* were upregulated during molting, which coincides with marking formation (Fig. 7). The result indicates that the common genes may be involved in marking formation in the three mutants.

**Fig. 8:**
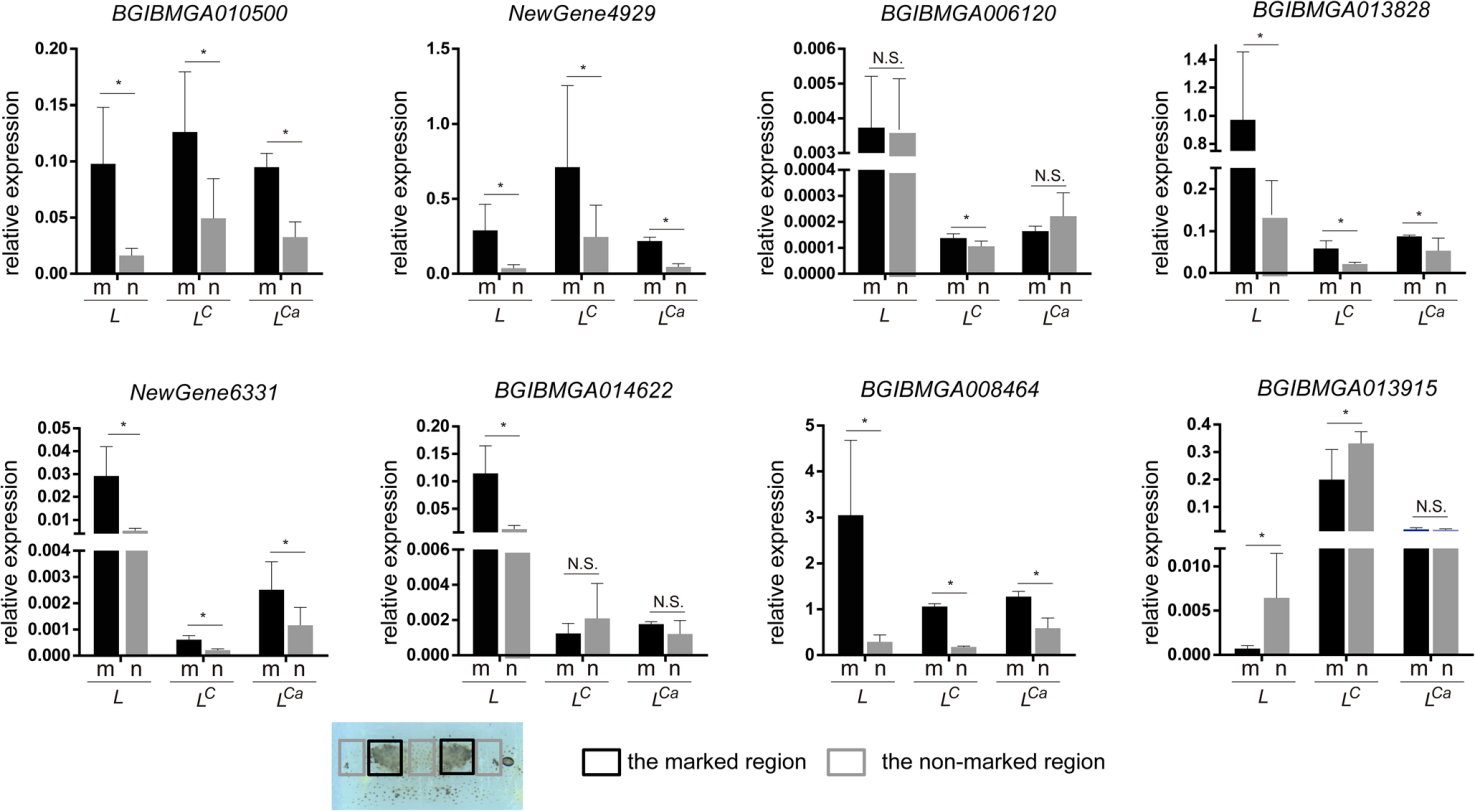
The eight common DEGs specifically expressed in the marking region of the three mutants. m, marking region; n, non-marking region. *P < 0.05, *n* = 3

Then, we detected the expression levels of the eight genes in pigmented and non-pigmented regions of the three mutants. *BGIBMGA010500*, *NewGene4929*, *BGIBMGA013828*, *NewGene6331*, and *BGIBMGA008464* are significantly upregulated in the pigment regions in all three mutants, *BGIBMGA006120* is only significantly upregulated in the pigmented regions of the *L*^*C*^ mutant, *BGIBMGA014622* is only significantly upregulated in the pigmented regions of the *L* mutant, and *BGIBMGA013915* is only significantly downregulated in the pigmented region of the *L*^*Ca*^ mutant (Fig. 8). These results further show that the identified common DEGs are closely related to mutant marking formation.

### Expression analysis of pigment genes

**Fig. 9:**
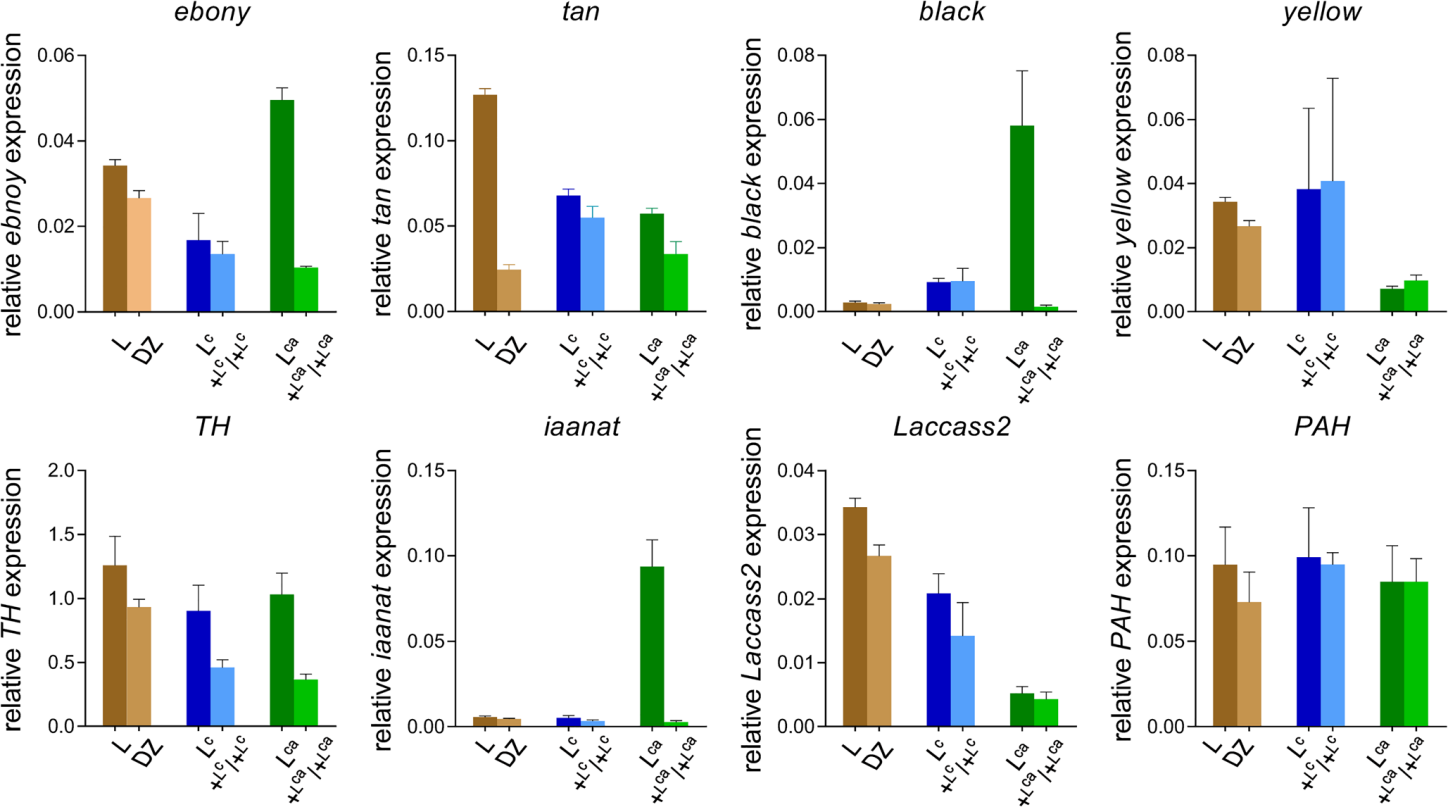
RT-qPCR analysis of major melanin biosynthesis pathway genes in the three mutants. Various-colored columns represent different strains

The phenotype of the three *L*-type mutants (*L*, *L*^*C*^, *L*^*Ca*^) includes twin-spots on the dorsal side of multiple segments. To investigate the basis of marking formation, we analyze the expression levels of major genes that participate in pigment synthesis. No significant change in expression levels was observed in most genes (Table 4). However, we performed RNA-seq of the integument of the mutants in HCS, whereas the expression of several major melanin synthesis–related genes expression is relatively very low. Previous studies in *Papilio xuthus* have shown that *PAH* is expressed throughout molting; *yellow* and *laccass2* are expressed in the middle of the molt stage; *Dat1*, *TH*, *DDC*, *ebony*, *tan*, and *black* are expressed later until ecdysis (Futahashi et al. 2010; Futahashi and Fujiwara 2005). Therefore, we detected the expression levels of *PAH*, *yellow*, *laccass2*, *Dat1*, *TH*, *ebony*, *tan*, and *black* in the integument of the mutants at 18 h after HCS through RT-qPCR (Fig. 9). In the three mutants, most of these genes are upregulated, indicating that melanin synthesis is aggravated, which coincides with the multi-markings phenotype. The selected stage to perform transcriptome analysis is HCS of the 4th instar, which occurs prior to melanin biosynthesis and marking formation. These findings suggest that the identified common DEGs are upstream regulators of pigment synthesis-related genes.

**Table 4:** Analysis of pigment biosynthesis genes expression in RNA-seq

Gene ID	Gene name	Fold change*		
		*L* VS DZ	*L*^***C***^ VS +^*LC*^/+^*LC*^	*L*^*Ca*^ VS +^*LCa*^/+^*LCa*^
Key genes for biosynthesis of melanin				
*BGIBMGA000563*	*TH*	− 0.9457	− 0.172695	0.652021
*BGIBMGA003199*	*Ddc*	0.500719	0.5696452	− 0.39646
*BGIBMGA003866*	*PAH*	0.331012	0.1114096	0.422383
*BGIBMGA008538*	*iAANAT*	0.022435	− 4.15E−05	− 0.08031
*BGIBMGA012088*	*black*	–	–	–
*BGIBMGA006740*	*laccase 2*	1.659908	0.4407536	2.150171
*BGIBMGA000031*	*ebony*	0.41412	− 0.063488	− 0.06704
*BGIBMGA002077*	*tan*	− 0.26242	− 0.052286	− 0.13387
*BGIBMGA001149*	*yellow*	0.595242	− 0.240408	− 1.85824
*BGIBMGA014032*	*yellow-f2*	− 1.36087	− 0.977823	− 0.96286
*BGIBMGA007253*	*yellow-e*	− 0.34174	− 0.127416	− 0.02174
*BGIBMGA003918*	*yellow-f4-2*	− 0.48026	− 0.725304	− 0.12375
*BGIBMGA007254*	*yellow-d*	− 0.64256	0.1928174	− 0.37524
*BGIBMGA007255*	*yellow-h2*	− 0.10514	0.2480949	0.363732
*BGIBMGA014224*	*yellow x*	0.006538	− 0.472216	− 0.09412
*BGIBMGA010917*	*yellow-f4*	− 0.35039	0.0255857	− 0.80856
Key genes for biosynthesis of ommochrome				
*BGIBMGA006740*	*Bmcardinal*	1.659908	0.4407536	2.150171
*BGIBMGA007285*	*BmGC*-1	− 0.2748	0.5931566	0.241481
*BGIBMGA007286*	*BmGC*-2	− 0.18631	0.2416701	− 0.24799
*BGIBMGA007856*	Kynurenine formamidase	0.767844	− 0.044082	0.700758
Key genes for biosynthesis of pteridine				
*BGIBMGA001235*	GTP-cyclohydrolase-a	0.230366	− 0.094524	0.105837
*BGIBMGA008134*	GTP-cyclohydrolase-b	–	–	−
*BGIBMGA003643*	PTPS	− 0.593526	− 0.069666	0.053928

*The expression is signification upregulated when the value of fold change is > 1 and signification downregulated when the value of fold change is < − 1

## Discussion

### *L*-type mutants are complex pigment mutants containing melanin and ommochrome

Our analysis revealed that the genes that participate in melanin synthesis are upregulated in mutants at 18 h after HCS, suggesting that melanin synthesis pathway may be activated (Fig. 9). In addition, we also found that *Bmcardinal*, which encodes a phenoxazinone synthetase that participates ommochrome synthesis (Osanai-Futahashi et al. 2016), is significantly upregulated in the *L* and *L*^*Ca*^ mutants (Table 4). Ommochrome is a group of insect pigments that mainly exist in insect eyes, eggs, and wings. It has also been reported that ommochrome are also produced in the epidermis of insects such as *Gryllus bimaculatus*, *Carausius morosus*, and *Mantis religiosa* (Linzen 1974). However, compared with the widely studied synthesis mechanism of epidermal melanin, the function and synthesis mechanism of ommochrome in the epidermis of insect larvae remains unclear. Studies have revealed that *quail* (*q*) and *quail-like* (*q-l*^*P*^) are complex pigment mutants that contain melanin, ommochrome, and pteridine, and *Bmcardinal* is also upregulated in the *q* and *q-l*^*P*^ mutants (Nie et al. 2014; Wang et al. 2017). Moreover, the absence of *Bmcardinal* activity results in suppressed red pigmentation around the eyespots of larval epidermis in the *p*^*S*^ strain (Osanai-Futahashi et al. 2016). Although *Bmcardinal* is upregulated in *q* and *q-l*^*P*^, the two mutants have no distinct eyespots and red pigmentation, and we infer that the upregulated *Bmcardinal* in *q*, *q-l*^*P*^, *L*, *L*^*C*^, and *L*^*Ca*^ results in different background skin colors compared with the wild type. In summary, the *L*-type mutants are complex pigment mutants that contain melanin and ommochrome and may be utilized in studying ommochrome pigments in insect larval epidermis.

### The identified DEGs are related to pigmentation in the three mutants

Except for the analysis of genes that are directly involved in pigment synthesis, we hope to identify novel genes that are relate to pigment synthesis and the establishment of the marking patterns in the three mutants via transcriptional analysis. We identified eight common DEGs in the three mutants. One of these is UDP-glycosyltransferase, which paralogous to the *Gb* locus (Daimon et al. 2010). Loss of UDP-glycosyltransferase in white cocoons prevents glycosylation of the 5-O position of dietary quercetin, thereby resulting in a significant decrease in total flavonoid content. To date, no study has on the role of UDP-glycosyltransferase in insect epidermal pigment synthesis has been conducted. This gene has also been identified in the butterfly wing color pattern transcriptome, suggesting that it might be involved in melanin formation (Zhang et al. 2017). In addition, some studies have shown that UDP-glycosyltransferase is involved in the formation of insect epidermis and tyrosine metabolism, and tyrosine is essential to melanin synthesis. We hypothesize that UDP-glycosyltransferase alters the state of melanin and/or melanin precursor substances and participate in the transport of these substances through glycosylation.

Fujiwara previously identified the *L* gene by positional cloning and reported that periodic expression of *wnt1* in response to ecdysteroid generates twin-spot markings of *L* (Yamaguchi et al. 2013). However, we note that the *Wnt1* and Wnt pathway genes were not included in the list of DEGs; the possible reasons is that the stage we performed RNA-seq is HCS, which is later than the stage of the upregulation of *Wnt1* gene in *L* mutant, and that we could not detect the differentially expressed between WT and the mutants. The other reason may be that *Wnt1* regulates body color formation in other ways which is independent of the Wnt signaling pathways, such as interaction with other signaling pathways. Previous studies have shown that the Wnt and Hedgehog signaling pathways both play roles in cell proliferation, differentiation, and embryo patterning in *Drosophila*, and the two signaling pathways have been proven to be not isolated. Some regulators have been identified to have the same effect on the Wnt and Hedgehog pathways, and crosstalk analysis identified hundreds of common proteins between the two pathways (Swarup et al. 2015; Toku et al. 2011). In this study, the *patched* gene (Table 3), a receptor of Hedgehog signaling, was significantly upregulated in the three mutants, suggesting that Hedgehog signaling participates in mutant phenotype construction. Network crosstalk analysis has indicate that the *patched* gene may be a node of the Wnt and Hedgehog signaling crosstalk networks (Toku et al. 2011). However, although studies involving butterfly revealed that the two pathways also participate in eyespots patterning (Keys et al. 1999; Tong et al. 2012), no study has shown that these interact with each other during this particular process. Our study suggests that the *L* mutant is an ideal material for investigation two important pathways and their interactions in relation to color pattern formation in insects.

There are three cuticular proteins in the eight common DEGs, which include two upregulated (*BGIBMGA010500* and *NewGene_4929*) and one downregulated (*BGIBMGA013915*) gene. The two genes, namely, *BGIBMGA010500* and *NewGene_4929*, may be the common substances for color-pattern formation in three mutants. Cuticular proteins constitute the main components of insect epidermis. Body color of insects depends on the content of pigment substances in the epidermis, and normal epidermis construction serves as basis for establishing body color pattern. Dai et al. found that the lack of a low-complexity epidermal protein *BmorCPH24* in silkworm causes abnormal markings, which suggests that coloration in silkworm larvae is influenced by cuticular proteins (Xiong et al. 2017). Previous transcriptomes analyses have been identified a number of cuticular proteins in the pigmented integument of silkworm strains, including *quail* (*q*) (Nie et al. 2014), *quail-like* (*q-l*^*p*^) (Wang et al. 2017), *black dilute* (*bd*) (Wu et al. 2016), sex-controlled melanism (*sml*) (He et al. 2016), and the epidermis of larvae and wings of adult in butterfly (Zhang et al. 2017), suggesting that cuticular proteins are important for epidermal construction and pigmentation. However, the identified cuticular proteins in these studies are largely different, although the same pigments occur in insects. We have two conjectures that may explain this discrepancy. (1) Various species employ different cuticular proteins that bind to the same pigment to form stable structures and/or (2) various developmental stages employ different cuticular proteins that interact with pigments. Further investigation on the underlying mechanism is thus warranted, which may also provide insights into the evolution of insects.

Except for the formation of the same marking pattern among the three mutants, fine regulation of different pigmentation ranges requires further investigation. According to the Venn diagram of DEGs (Fig. 6), there are 257 specific DEGs in *L*, 43 specific DEGs in *L*^*C*^, and 114 specific DEGs in *L*^*Ca*^. We hypothesize that the specific DEGs may be related to the fine regulation of different pigmentation ranges among the three mutants. However, how the identified common DEGs participate in marking pattern formation among the three mutants and which genes are involved in the fine regulation of the different pigment ranges require further studies.

## Conclusions

In this study, we analyzed the transcriptome of integuments of three allelic multiple twin-spot markings mutants and identified a number of DEGs, which were further subjected to GO and KEGG enrichment analyses. We observed that the genes involved in the biosynthesis of melanin and ommochrome were upregulated in the three mutants, suggesting their role in the generation of markings. We also identified common DEGs in the three mutants that may act as upstream regulators of pigment synthesis-related genes. The common DEGs are upregulated during molting, which coincide with marking formation and are significantly differentially expressed between marking and non-marking regions, suggesting that the common DEGs are involved in the formation of the *L*-type marking pattern. Except for the common DEGs, we have identified specific DEGs in the three mutants, which may be related to the fine regulation of the different pigmentation ranges in the three mutants. This research provides a reference for understanding of the genetic basis of *L*-type mutants.

## Materials and methods

### Silkworm strains

The silkworm strains used in this study were obtained from the Silkworm Gene Bank in Southwest University in Chongqing, China. The larvae were fed fresh mulberry leaves under standard conditions with 75% relative humidity at 25 ± 2 °C. *L* and Dazao were used in a pairwise comparison; *L* is an inbred line of the Dazao that was obtained after years of crossing. *L*^*C*^/+^*Lc*^ and +^*Lc*^/ +^*Lc*^, and *L*^*Ca*^/+^*LCa*^ and +^*LCa*^/+^*LCa*^ were also used in pairwise comparisons.

### cDNA library preparation and Illumina RNA-seq

The integuments were dissected from four individuals of each strain on HCS of the 4th instar; fat body and the trachea, which adhere to the integument, were removed using tweezers. The samples were stored at − 80 °C in TRIzol reagent after quick freezing with liquid nitrogen. The dissections are conducted in a systematic manner between different groups to reduce experimental error. Total RNA of each mixture, which contains the pooled integuments of four individuals, was isolated using TRIzol reagent (Invitrogen, Carlsbad, CA, USA) according to manufacturer’s instructions. RNA purity and concentration were assessed using a NanoDrop ND-2000C spectrophotometer (Thermo, Waltham, MA, USA). RNA integrity was assessed using the Agilent Bioanalyzer 2100 system (Agilent Technologies, Palo Alto, CA, USA). Then, mRNA enriched and fragment interruption, cDNA synthesis, the addition of adapters, PCR amplification, and Illumina sequencing were performed by Beijing Biomarker Technologies (Beijing, China).

### Data analysis

Raw reads from the Illumina HiSeq, which are based on the sequencing by synthesis (SBS) technology, were evaluated using the Phred formula (Ewing et al. 1998). Low-quality reads including *N* with a ratio > 10% and the number of the bases *Q* ≤ 10 that accounts for > 50% of the whole reads were ruled out to obtain high-quality clean data. Then, the clean data were mapped to the silkworm reference genome (ftp://ftp.ensemblgenomes.org/pub/metazoa/release-37/fasta/bombyx_mori/) using TopHat2 (Kim et al. 2013), which is based on the alignment software Bowtie2 (Langmead et al. 2009). Aligned efficiency (mapped reads accounts for the percentage of clean reads) was calculated to evaluate utilization of the transcriptome data. Cuffquant and Cuffnorm used FPKM (Florea et al. 2013) as indicator of the level of transcriptional or gene expression, with FPKM = cDNA fragments/mapped fragments (millions) × transcript length (kb). Differential expression analysis was performed based on the FPKM of genes with a threshold *P* < 0.05. Functional annotations of DEGs were analyzed using BLAST (Altschul et al. 1997) and HMMER. GOseq and KOBAS2.0 were used to perform GO and KEGG enrichment analyses of differentially expressed transcripts, respectively.

### Identification and functional annotation of new genes

Cufflinks software (Roberts et al. 2011) was used to assemble the mapped reads and compare with the reference genome annotation information to identify novel transcripts and genes. The transcripts without corresponding templates in the reference genome annotation information were defined as new transcripts and new genes. The new genes were blast in NR, GO, Swiss-Prot, COG, KOG, and KEGG through blastX. The amino acid sequences of the new genes were predicted and then blast in Pfam with HMMER [136] software to obtain annotation information of the new genes.

### RT-qPCR

The primers used for RT-qPCR were selected from qPrimerDB (Lu et al. 2018). RT-qPCR was performed on a CFX96™ Real-Time PCR Detection System (Bio-Rad, Hercules, CA USA) with a SYBR Green RT-qPCR Mix (Bio-Rad). The PCR conditions were as follows: 95 °C for 3 min followed by 40 cycles of 95 °C for 10 s and 60 °C for 30 s. Three biological repeats were performed for each genotype, and each sample was analyzed in triplicate to rule out technical errors. Relative expression was calculated using the 2^−ΔΔCt^ method (Livak and Schmittgen 2001) with *B. mori eukaryotic translation initiation factor 4A* as reference.

### Microarray analysis of spatiotemporal gene expression

Based on microarray data for gene expression in silkworm larva (data unpublished), we analyzed the expression patterns of the eight common DEGs. Two of these genes were newly identified transcripts in RNA-seq; there is no corresponding microarray data. Microarray data of the remaining six genes were processed as previously reported (Xia et al. 2007; Zhang et al. 2012).

## Electronic supplementary material

ESM 1 (XLSX 159 kb)

ESM 2 (XLSX 2619 kb)

## Data Availability

The datasets generated in this study are publicly available at NCBI–SRA (www.ncbi.nlm.nih.gov/sra) accession: SRR7894599, SRR7894600, SRR7894601, SRR7894602, SRR7894603, SRR7894604, SRR7894605, SRR7894606, SRR7894607, SRR7894608, SRR7894609, SRR7894610, SRR7894611, SRR7894612, SRR7894613, SRR7894614, SRR7894615, SRR7894616.
